# Editorial: Methods in brain stimulation

**DOI:** 10.3389/fnhum.2025.1619784

**Published:** 2025-05-28

**Authors:** Tad T. Brunyé, Frigyes Samuel Racz

**Affiliations:** ^1^Cognitive Science and Applications Branch, U.S. Army DEVCOM Soldier Center, Natick, MA, United States; ^2^Center for Applied Brain and Cognitive Sciences, Tufts University, Medford, MA, United States; ^3^Department of Neurology, Dell Medical School, The University of Texas at Austin, Austin, TX, United States

**Keywords:** non-invasive brain stimulation, neuromodulation methods, personalized stimulation, stimulation protocols, transcranial electrical stimulation

## 1 Introduction

Non-invasive brain stimulation (NIBS) research is expanding at a rapid pace, driven by the promise of reliably altering cognition, emotion, and motor behavior across research, clinical, and other applied domains. The recent proliferation of electrical, magnetic, photonic, and ultrasonic brain stimulation approaches demonstrates an evolution toward an increasingly versatile toolbox for researchers and clinicians. However, this rapid technological progress has often outpaced the establishment of best practices, standardized protocols, and mechanistic understandings. The Methods in Brain Stimulation Research Topic was launched to help fill this knowledge gap.

## 2 Article contributions

There were eight contributions to this Research Topic, most of which showed a strong emphasis on expanding and refining stimulation methodologies to meet clinical and applied demands. Hanlon et al. present a comprehensive review of a two-stage bilateral deep transcranial magnetic stimulation (TMS) protocol for Parkinson's disease, combining motor and prefrontal cortical stimulation to address both motor and non-motor symptoms. Their findings highlight not only moderate therapeutic efficacy but also the need for further investigation into dosing and durability. In a parallel effort to map and systematize the field, Liu, Luo, et al. offer a scientometric review of transcranial alternating current stimulation (tACS), tracking its rapid expansion over the past decade. Their analysis charts global trends and underscores a rising interest in oscillatory entrainment and applications to neurodegenerative diseases such as Alzheimer's Disease. Together, these contributions underscore a growing maturity in both the therapeutic ambition and methodological precision of the brain stimulation field.

Several contributions grapple directly with the challenge of optimization, including how to best configure stimulation parameters for reliable, domain-specific outcomes. Santander et al. conduct a sweeping meta-regression across five cognitive domains, identifying within-subjects designs as more reliable and pointing to nuanced effects of stimulation polarity. Duffy et al. complement this work by examining tDCS outcomes in active-duty Soldiers, illustrating not only enhancement of executive function and attention, but also potential tradeoffs such as increased risk-taking and reduced working memory, raising real-world implications for safety and mission readiness. Toth et al. further contextualize these findings in their conceptual overview, emphasizing five critical methodological pursuits, from closed-loop systems to bias reduction and improved dose-response modeling. These works collectively advocate for more tailored, context-aware approaches to stimulation design and evaluation in order to reliably establish causal inferences between brain function, NIBS and behavioral outcomes.

Importantly, precision and personalization emerge as central themes across multiple contributions to this Research Topic. Liu, Sundman, et al. compare functional- and structural-connectivity guided repetitive TMS targeting and find greater reproducibility with structural methods, providing key support for connectome-informed protocols. Van der Groen et al. reinforce the need for context-specific validation in their systematic review of electrical stimulation in military settings, highlighting variable effects and methodological inconsistencies across real-world tasks. Finally, Alipour et al. look beyond conventional methods, reviewing magnetothermal neuromodulation as a next-generation approach with high spatial precision and minimal invasiveness). Their contribution widens the scope of this Research Topic by exploring the biophysical underpinnings and clinical promise of magnetic field-based techniques. Across these studies, we see a clear push toward greater specificity, safety, and scalability in brain stimulation methods. There is also an emergent call for, as well as laying the foundation for, a new era of individualized, mechanism-driven neuromodulation.

## 3 Integrative themes and future directions

Articles published in this Research Topic used diverse methodological approaches including scientometric mapping, meta-analysis, connectome-informed targeting, and dual-domain protocols. Notably, five out of eight publications are review or systematic review articles, providing a wide coverage and synthesis of contemporary NIBS literature. At least three themes emerged from these efforts.

First, several contributions highlight an emergent shift from “one-size-fits-all” stimulation to personalized approaches. These include Liu, Sundman, et al. connectome-based targeting work, Toth et al. call for individualized protocols, and Santander et al. meta-analytic evidence for domain- and design-specific effects. Second, there is a complementarity between conceptual and empirical work. The theoretical reflections of Toth et al. complement the data-driven explorations in other articles (Duffy et al.; Hanlon et al.; Santander et al.), providing context for understanding the complexity of dose-response relationships and inter-individual variability. Third, there are common calls for increased standardization and replication. Multiple articles point to a lack of harmonized protocols, consistent outcome measures, and adequately powered samples that limit field-wide synthesis and result in slow progress (Liu, Luo, et al.; Santander et al.; Van der Groen et al.).

Looking ahead, several priority directions emerge for advancing methods in brain stimulation research. These include:

- Parameter optimization frameworks leveraging multivariate and adaptive modeling to fine-tune stimulation settings.
- Standardized outcome metrics to enable cross-study comparisons of efficacy and safety across diverse domains and populations.
- Closed-loop and adaptive systems that dynamically adjust stimulation based on neural or behavioral feedback.
- Greater integration of neuroimaging, electrophysiology, and computational modeling to capture stimulation effects across spatial and temporal scales.
- Population- and context-specific validation, especially for applied groups such as military personnel, clinical populations, and older adults.
- Ethical and regulatory considerations and continued dialogue among researchers, clinicians, ethicists, and policy makers to guide responsible NIBS development and deployment.
- Emphasis on establishing causal inferences between intervention and outcome by better understanding the neurobiological effects of various NIBS modalities.

## 4 Conclusion

This Research Topic highlighted the importance of methodological innovation, transparency, and rigor in brain stimulation research. Together, the eight contributions offer empirical evidence, conceptual clarity, and forward-looking frameworks that will inform the next generation of NIBS efforts. As brain stimulation becomes increasingly integrated into research, clinical practice, and other professional and recreational applications, method-focused work such as this becomes vital to ensuring maximal benefits and minimal harm.

